# A novel compound heterozygous *COL4A4* mutation in a Chinese family with Alport syndrome: A care case report

**DOI:** 10.1097/MD.0000000000027890

**Published:** 2021-11-24

**Authors:** Ji-Yu Chen, Jing-Jing Cui, Xi-Ran Yang, Yan-Fang Li, Yan-Hua Zhang, Jia-Ni Chen, Jun-Yu Lin, Bo Zhao

**Affiliations:** a Department of Nephrology & Rheumatology, Kunming Children's Hospital, Kunming, Yunnan, China; b Dali University, Dali, Yunnan, China.

**Keywords:** Alport syndrome, *COL4A4*, genetics, triple compound heterozygous

## Abstract

**Rationale:**

Alport syndrome (AS) is an inherited progressive renal failure, characterized by kidney disease, hearing loss, and eye abnormalities.

**Patient concerns:**

A 7-year-old male child was admitted for persistent microscopic hematuria and proteinuria.

**Diagnoses:**

Combined with clinical manifestations, laboratory testing, pathological changes of kidney and sequencing results, the patient was diagnosed as AS.

**Interventions:**

The patient was treated with ACEI and tacrolimus drugs for 2 years, but continued to have hematuria and proteinuria. Thus, a genetic analysis was performed using next-generation sequencing in four affected members from the family.

**Outcomes:**

The findings revealed triple compound heterozygous mutation of *COL4A4*: three novel variations, c.1045C>T (p. R349X), c.3505+1G>A (splicing), and c.2165G>A (p. G722D).

**Lessons:**

This study was novel in finding that a triple variant of the *COL4A4* gene simultaneously in trans and in cis. The effects of multiple mutation sites and the type of gene mutation in AS were also underlined.

## 1 Introduction

Alport syndrome (AS) is an inherited progressive disease caused by mutations in the type IV collagen gene encoding for the α3, α4, and α5 chains. It is clinically characterized by persistent hematuria, proteinuria, and end-stage renal disease (ESRD), accompanied by sensorineural hearing loss and ocular abnormalities.^[1]^ The incidence of the disease was estimated to be approximately 1 in 5000 in the United States, accounting for 0.5% of the new-onset patients with ESRD in adults, about 3% of children, and 1% to 2% of patients with ESRD in Europe.^[2,3]^ At present, no effective approach to the radical treatment of AS is available. Moreover, heterozygous individuals at risk of renal failure are often underdiagnosed.^[4]^ With the development of next-generation sequencing (NGS) technology, rare AS gene variations have been discovered. Over the past several years, some investigators have reported the existence of dysgenic inheritance in AS and even more complex combinations of variants of type IV collagen genes.^[5]^ Recently, the complex combinations of variants of type IV collagen genes have come into focus. For example, mutations in *NPHS2*, *LAMA5*, and *MYO1E* genes can also affect the phenotype of patients who have at least 1 mutation in a type IV collagen gene.^[6–8]^ Hence, these newly discovered combinations of atypical mutations present new challenges to genetic researchers and clinicians.

To our knowledge, few studies have reported on patients with AS caused by *COL4A4* genetic mutation in trans and in cis. This study aimed to describe a rare family of AS in the Bai minority ethnic population. Three novel heterozygous mutations in the *COL4A4* gene, which is an uncommon compound heterozygous mutation of *COL4A4*, were found in the family.

## 2 Case presentation

**Figure 1:**
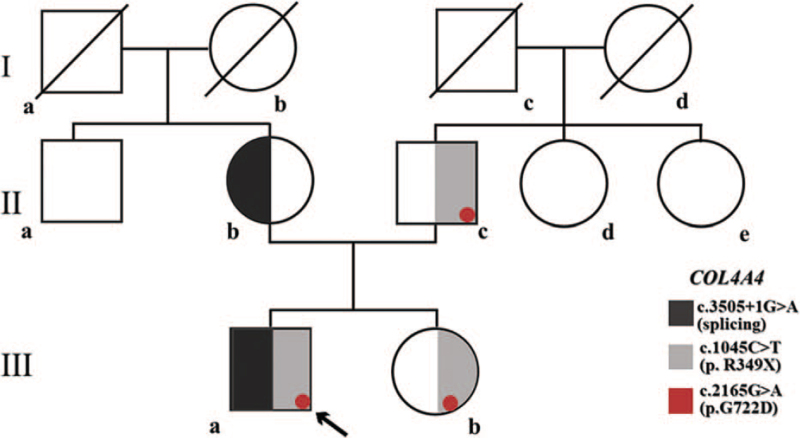
A 3-generation pedigree of the patient's family.

A 7-year-old male child presented to a community hospital with frequent nocturnal enuresis of two weeks duration. At that time, the laboratory finding showed that he suffered from persistent microscopic hematuria and proteinuria. A review of systems was negative for fevers, cough, sore throat, rash, edema of both lower limbs, any dimorphic syndrome, or a history of urinary tract infection or joint pain. He had a significant familial history of kidney disease. His maternal grandmother died at age 65 years of ESRD. His 26-year-old mother reported hematuria and proteinuria since she was a teenager. Moreover, his father and his sister presented with intermittent microscopic hematuria as well. There was family history of similar hematuria in other family members, including his 2 aunts and 1 uncle (Fig. 1).

On physical examination, the patient's heart rate was 96 beats per minute, blood pressure was 103/63 mmHg, and respiratory rate was 23 breaths per minute, without pulmonary rales, arrhythmia, edema of the eyelids and lower limbs, and percussive pain in both kidneys. Furthermore, the neurological examination was unremarkable.

The biochemical indices of the patient are summarized in Table 1. The patient's immune-related antibodies were negative, which included anti-nuclear antibodies, anti-Sm antibodies, anti-neutrophil cytoplasmic antibodies (ANCA), and so forth. When examined for hematological parameters, the leukocyte count (6.33 × 10^9^/L), lymphocyte count (2.77 × 10^9^/L), neutrophil count (3.07 × 10^9^/L), red blood cell count (4.61 × 10^12^/L), hemoglobin (127 g/L), platelet count, and serum C-reactive protein level were normal. Other laboratory test results were negative for hepatitis B virus, hepatitis C virus, human immunodeficiency virus, and syphilis. Renal ultrasound examinations, fundus examination, and otoacoustic emission inspection were normal.

**Table 1:** Laboratory examination results of the patient.

Items	Results	References
Albumin	31.6 g/L	35—50 g/L
Total protein	52.0 g/L	60–80 g/L
Creatinine	27 μmol/L	27—62 μmol/L
Urea nitrogen	5.3 μmol/L	1.8–6.4 μmol/L
Total cholesterol	5.2 mmol/L	3.12–5.2 mmol/L
Triglyceride	0.8 mmol/L	0.8–1.8 mmol/L
Complement C3	1.24 g/L	0.8–1.5 g/L
Complement C4	0.23 g/L	0.12–0.4 g/L
Urinary protein	3+	Negative
24-h Urine protein	1.09 g/24 h	0–0.15 g/24 h
Urine red cell	30–40/HPF	0–3/HPF

**Figure 2:**
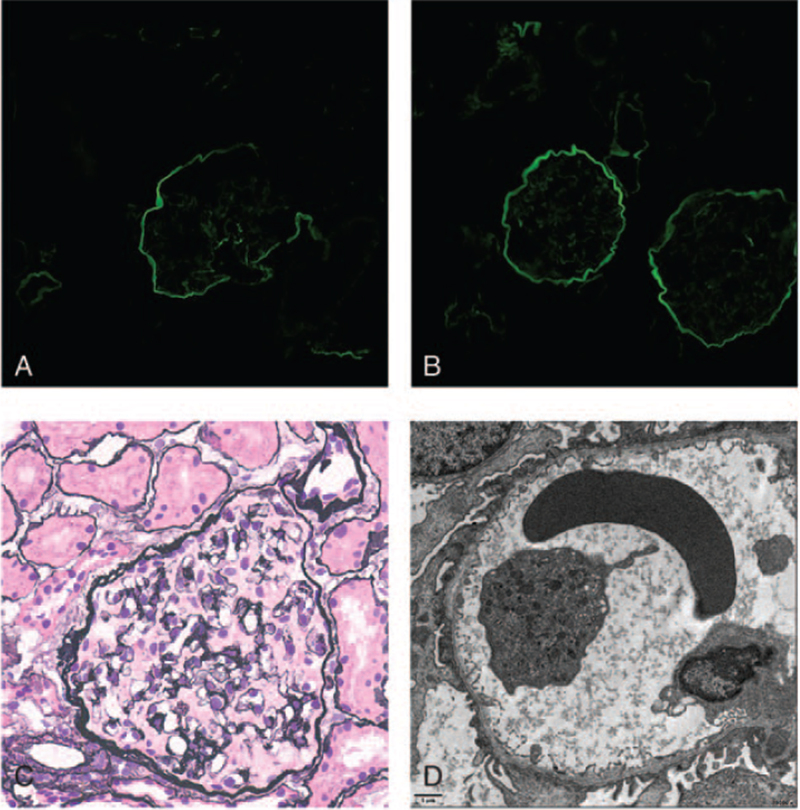
Results of renal biopsy. (A) Immunofluorescence staining of IV staining collagen α3 in renal tissue from the patient (×400). Panel A shows the antibody for α3α4α5 heterotrimer stating the glomerular basement membrane (GBM) is negative. (B) Immunofluorescence staining of IV staining collagen α5 in renal tissue from the patient (×400). Panel B shows the bowman's capsular and tubule BMs are labeled but the GBM is unstained of the α5. (C) Images of renal pathology under light microscopy by periodic acid-silver metheramine staining (×400). (D) Irregular GBM thinning, thickening, and splitting in the electron micrograph of renal tissue (×6000).

The kidney biopsy specimen showed pathological changes compatible with AS. Immunofluorescence staining showed staining for α3 and α5 in glomerular basement membrane (GBM) was absent (Fig. 2A and B). Glomerulosclerosis and segmental glomerulosclerosis were not otherwise identified in PASM (periodic acid-silver metheramine) -stained kidney sections (Fig. 2C). The ultramicroscopic evaluation revealed that segments of the GBM exhibited irregular thinning and thickening or splitting, and fusion of the most foot process (Fig. 2D). The patient was treated with prednisone (1 mg/kg/day) for 2 weeks, ACEI, and tacrolimus (0.05 mg/kg/day) drugs for 2 years, but continued to have hematuria and proteinuria during follow-up.

The persistent hematuria, proteinuria, and pathological changes of kidney were consistent with a diagnosis of AS. NGS was performed on the genomic DNA of the patient, his parents, and a sister to investigate the patient condition at the genetic level.

## 3 Genetic investigation-method

Thus, after acquiring the informed consent of both parents and the Ethics Committee of Kunming Children's Hospital, all exons of 506 genes associated with urinary system diseases were analyzed using NGS sequencing performed by MyGenostics, Inc. (Beijing, China), which is CAP-accredited. After sequencing, the bioinformatics analysis was used to analyze the raw data and to identify the sites of mutation by comparing the DNA sequences with the corresponding GenBank (https://www.ncbi.nlm.nih.gov/) reference sequences. Finally, we used Sanger sequencing to confirm all identified mutations. Besides, genomic DNA from all available family members was also obtained for Sanger sequencing.

## 4 Genetic investigation results

**Figure 3:**
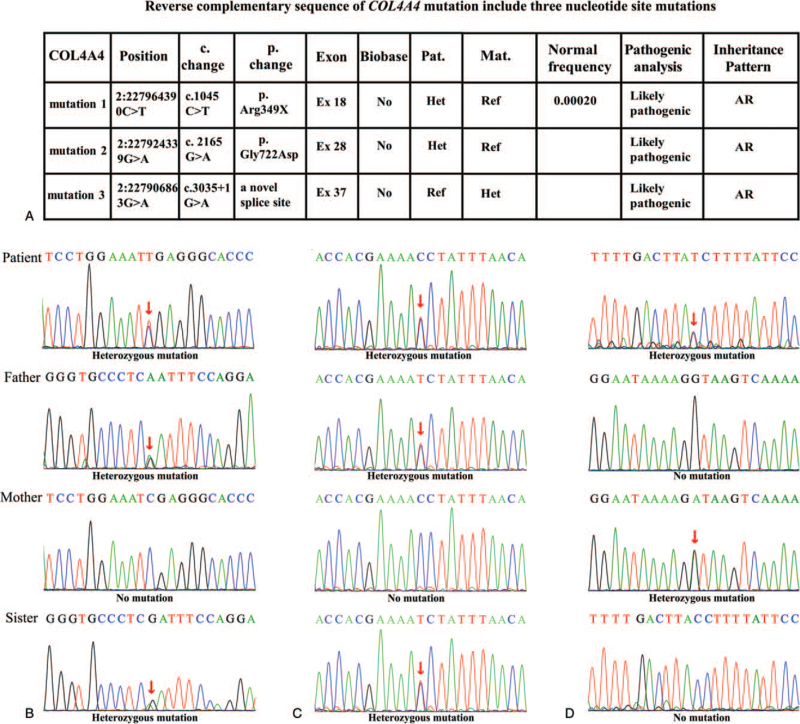
Genotype and conservation of the patient's mutations in *COL4A4*. (A) Table with Chromosome position, c. change, p. change, exon number, normal frequency, pathogenic analysis, and inheritance pattern in our patient. (B–D) Sanger tracing for the patient, the mother, the father, and sister for each allele.

The genetic investigation results (Fig. 3A) revealed that the patient carried three novel heterozygous mutations and a known variation in *COL4A4*, which was a rare mutation combination. These mutations were validated by Sanger sequencing (Fig. 3B–D). The c.3505+1G>A(splicing) variant was inherited from his mother (Fig. 3A). The frequency of the variant allele in this population was close to 0. The variant did not appear in the frequency of the ESP6500si (http://evs.gs.washington.edu/EVS/), dbSNP (https://www.ncbi.nlm.nih.gov/projects/SNP/), and 1000g2015aug_all database (http://browser.1000genomes.org). Based on these data, the variant was classified as “Likely Pathogenic” according to the American College of Medical Genetics and Genomics (ACMG) guidelines. The other c.1045C>T (p. R349X) and c.2165G>A (p. G722D) variants were inherited from his father (Fig. 3A). The frequency of these variant alleles in this population was 0.00020 and close to 0, respectively. The c.1045C>T (p. R349X) and c.2165G>A (p. G722D) variants were also not found in the Human Genome Mutation Database (http://www.hgmd.org/).

## 5 Discussion

The present study provided further evidence for complicated genotype in AS. The three novel *COL4A4* mutations were observed in the patient diagnosed with AS, as an atypical compound heterozygous mutation. Moreover, this genotype has not been reported so far in the literature.

In this study, a 7-year-old male patient presented with persistent hematuria, proteinuria, and pathologic changes compatible with AS. Genetic examination revealed that a mutation in *COL4A4* might be the cause of AS. Two c.1045 C>T (p. R349X) and c.2165G>A (p.G722D) mutations, which formed a compound heterozygous mutation with c.3505+1G>A (splicing), were found in this study. The combination of a triple pathogenic variant in *COL4A4* is rare.

**Figure 4:**
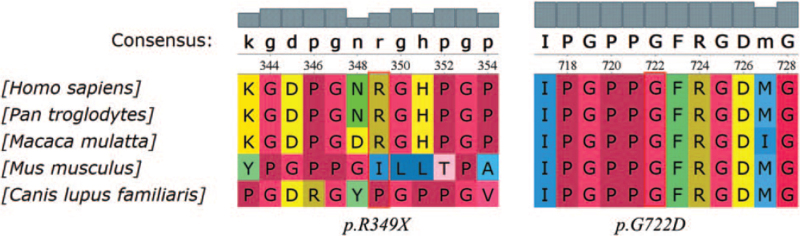
Phylogenetic comparisons of amino acid sequences of *COL4A4* protein regions affected by the identified mutations.

Further, the conservation status of these exonic variants was checked using a unified bioinformatics toolkit (UGENE) software in 5 species: *Homo sapiens*, *Pan troglodytes*, *Macaca mulatta*, *Mus musculus*, and *Canis lupus familiaris*. The findings for *P troglodyte* and *M mulatta* showed that c.2165G>A (p.G722D) and c.1045C>T (p.R349X) variants were conservative in primates, suggesting the possible pathogenicity in human beings (Fig. 4).

The present study showed that it is the first time to report the *COL4A4* mutations in trans and cis. The co-inheritance of 2 *COL4A4* mutations in trans have been inherited from one of paternal and one of maternal origin, which is called “autosomal recessive AS (ARAS).” Moreover, there is a second mutation in cis in the paternal allele. Therefore, this patient fulfills the criteria for diagnosing ARAS but has the uniqueness of carrying a third *COL4A4* mutation. Presently, genetic models for AS were as follows: autosomal inheritance with mutations in trans resembling the risk of a recessive disease; autosomal inheritance with mutations in cis resembling the risk of a dominant disease. In 1997, researchers reviewed the clinical spectrum associated with mutations of several chains of type IV collagen.^[9]^ They listed the three mutations that had been identified in the *COL4A4* gene: 1 in familial benign hematuria and 2 in AS with the development of renal failure at 14 and 18 years of age, respectively. Moreover, fewer mutations were described for recessive than for X-linked disease, and too few were known for genotype–phenotype correlations.^[10]^ A previous study reported the impact of common genetic variants on gene expression in 869 individuals and discovered that the expression of many genes is affected by common variants in cis or in trans. Furthermore, it shows that variants affecting gene expression in cis often affect gene expression in trans.^[11]^

In the present study, the patient had 3 heterozygous mutations in the *COL4A4* gene. A previous study reported on 2 mutated alleles at the *COL4A4* locus in addition to 1 mutated allele at the *COL4A5* locus.^[5]^ Compared with that, the present study reported three mutated alleles at a unique single *COL4A4* locus. The present study was the first to report the *COL4A4* gene having “many” compound heterozygous mutations in AS. The reported data showed that the triple compound heterozygous proband exhibited earlier onset and a more severe clinical phenotype than the patient's 1 or 2 heterozygous relatives. This might imply that this extra mutation confers the risk of a more accelerated ARAS phenotype. In addition, the clinical phenotype of the patient's mother was found to be more severe than that of the father and sister by pedigree analysis. Previous studies found a similar phenomenon that affected participants with splice mutations or truncating mutations, in which each participant had a younger age at the onset of ESRD, compared with the age of participants with missense mutations.^[12]^ It was demonstrated that splicing mutations of *COL4A5* that created a premature stop codon and a truncated transcript were associated with a worse prognosis. This might partly explain the results. However, more relevant cases need to be studied to investigate whether a genotype–phenotype correlation exists in *COL4A4*-relate ARAS or autosomal dominant AS. Moreover, some studies suggested that *COL4A4* heterozygous mutation-associated type IV collagen kidney disease resulted in heterogeneous phenotypes. Thus, the effects of multiple mutation sites in combination with modifier genes and other factors should be considered.

In summary, three novel variations, c.1045C>T (p. R349X), c.3505+1G>A (splicing), and c.2165G>A (p. G722D), in the *COL4A4* gene were identified and were first found simultaneously in trans and cis. Therefore, the findings of this study might contribute to improving disease diagnosis and extending the mutational spectrum of AS.

## Author contributions

All authors read and approved the final manuscript.

**Conceptualization:** Ji-Yu Chen, Bo Zhao.

**Formal analysis:** Ji-Yu Chen, Jing-Jing Cui.

**Methodology:** Xi-Ran Yang, Yan-Fang Li.

**Resources:** Yan-Hua Zhang, Jia-Ni Chen, Jun-Yu Lin.

**Software:** Ji-Yu Chen.

**Supervision:** Bo Zhao.

**Validation:** Yan-Hua Zhang.

**Writing – original draft:** Ji-Yu Chen.

**Writing – review & editing:** Ji-Yu Chen, Bo Zhao.
